# ‘Remediation of pre-clerkship clinical skill performance using a hybrid coaching model’

**DOI:** 10.1080/10872981.2020.1842660

**Published:** 2020-10-30

**Authors:** Sarah Yonder, Jyotsna Pandey

**Affiliations:** a Assistant Professor of Family Medicine, Central Michigan University School of Medicine, Mt. Pleasant, MI, USA; b Professor of Pathology, Central Michigan University School of Medicine, Mt. Pleasant, MI, USA

**Keywords:** Clinical skills, remediation, self-regulated learning, co-regulated learning, deliberate practice, clinical skills exam performance

## Abstract

Substance

We reviewed the effect of a hybrid remediation model combining co-regulated learning and deliberate practice on future exam performance of pre-clerkship medical students who had been unsuccessful on a previous clinic skills exam. With this remediation model, we aimed to strengthen students’ self-regulated learning to improve future exam performance and support sustained and improved learning. **Educational problem addressed**: Observing that some students who initially performed well post remediation with deliberate practice, still struggled on future exams, we looked to address a method that could improve both short-and long-term clinical skills learning success with sustained performance. **Intervention outcome**: Comparing the remediated students’ exam scores pre- and post-coaching to their cohort’s performance, we observed the majority of students post remediation performed above their cohort’s exam average. **Lessons learned**: Combining learning models resulted in improved learning outcomes.

## Substance of innovation

Remediating clinical skills exam performance is a necessary and important part of pre-clerkship clinical skills teaching. Many learning models have been touted in medical education and use of some in remediating clinical skills exam performance has been evaluated. Only a few models have been shown to benefit the medical learner. For example, it has been noted that students’ use of self-regulated learning (SRL) improves outcomes as compared to those that do not use it [1]. Students, however, have variable strengths and weaknesses, so need to be remediated with an individualized strategy using a standards-based approach. Our aim for remediation was to focus on building skills and their retention to improve future performance.

Several studies applied learning models to remediate clerkship students’ clinical exam with success [2,3]. However, we have not found literature for pre-clerkship years utilizing models that promote self-awareness and SRL for future skills acquisition. With the aim to institute a remediation plan that both ensures desired outcomes for the current skills deficit and strengthens SRL for future, we tested a self-regulated learning model based on a hybrid of two frameworks (a) deliberate practice (DP) and (b) co-regulated learning (CRL). The hybrid coaching model was utilized to remediate students who did not pass a performance-based clinical skills exam. Through DP we gave direct feedback on their skills, and through CRL, we built and improved their own SRL approach to enhance future performance. This approach aids in self-identification of areas to improve and develop self-remediation in future for improved skills.

The use of DP in our model promotes recognition of areas of difficulty and self-regulated learning. Looking to also foster students’ self-sufficiency in future learning we propose the application of co-regulated learning to enhance performance and students’ engagement in ongoing instruction. The hybrid framework helps the pupil to adapt to the learning needs and promote self-motivated recognition of areas of improvement. Additional coaching is provided to enhance the ability for self-evaluative judgment and promote self-support for a sustained improvement and SRL. DP promotes performance improvement with a focused approach on recognizing areas of difficulty utilizing a teacher/coach and a standards-based practice of those skills to improve performance [4]. While DP has been used by athletes and musicians to enhance performance, it has also been applied in medical education for teaching and remediating clinical reasoning, including successful remediation of students’ skills performance [2,5]. Previously we utilized DP to remediate students after a poor exam performance. Occasionally students would continue under performing on subsequent skills exams. We began to review other learning theories to apply in conjunction with DP to enhance SRL skills. SRL involves goal setting and use of strategies to obtain goals, as well as monitoring as one works toward them. Over time students reflect and make changes as needed [6]. With this in mind, SLR is a powerful concept for medical students to enhance learning [7]. However, not all medical students are self-regulated learners or use SRL to their advantage [1]. Therefore, we hypothesized that the addition of a CRL approach, in combination with DP will be useful. Co-regulated learning involves learner and teacher interaction that enhances the learning process, and can support the student’s SRL as the teacher and environment play a role [8]. CRL has been suggested as a model for graduate medical education with resident learners and supervising faculty; in a clinical environment, less experienced trainees work with senior residents and faculty who support and enhance trainee learning and SRL [9].

Our clinical skills exams consist of three skill stations. Students are required to pass each station to pass the exam. The remediation process involves the course director (CD) informing the student about the station(s) he/she did not pass. Two remediation sessions follow: the first session involves a CRL approach, where the CD reviews areas of poor performance, answers questions, and emphasizes learning strategies such as planning, monitoring, and reflecting, tenets of SRL. In CRL fashion, faculty and student partner as the faculty steers student progression with focused feedback. As seen in work by Bransen and colleagues with clerkship students, faculty interact with students in clinical work, asking questions and providing direction, helping students see weak or ‘gap’ areas where they need to redirect their learning [10]. The next session takes a DP approach, by continuing to support a student’s SRL, encouraging reflection on skill acquisition and readjusting as he/she moves forward. Students can see areas to focus on and direct their study accordingly. The average time for each session is an hour. In combining CRL and DP, our approach promotes SRL in students remediating clinical skills.

## Educational problem it addresses

We recognized the need to enhance and strengthen student SRL in pre-clerkship clinical skills utilizing a standards-based model. The hybrid remediation model was developed as we felt that DP alone was insufficient for both short-and long-term clinical skills learning success and sustained performance. Students’ failure to get a passing grade on an exam helped us to identify those with weaker skills. Using our intervention of a hybrid remediation model, we equip them with a better strategy to learn and practice skills as they move ahead. In this way, we can help students recognize subpar learning methods, and have them retool for improved learning.

## Intervention outcome measured

**Figure 1.**
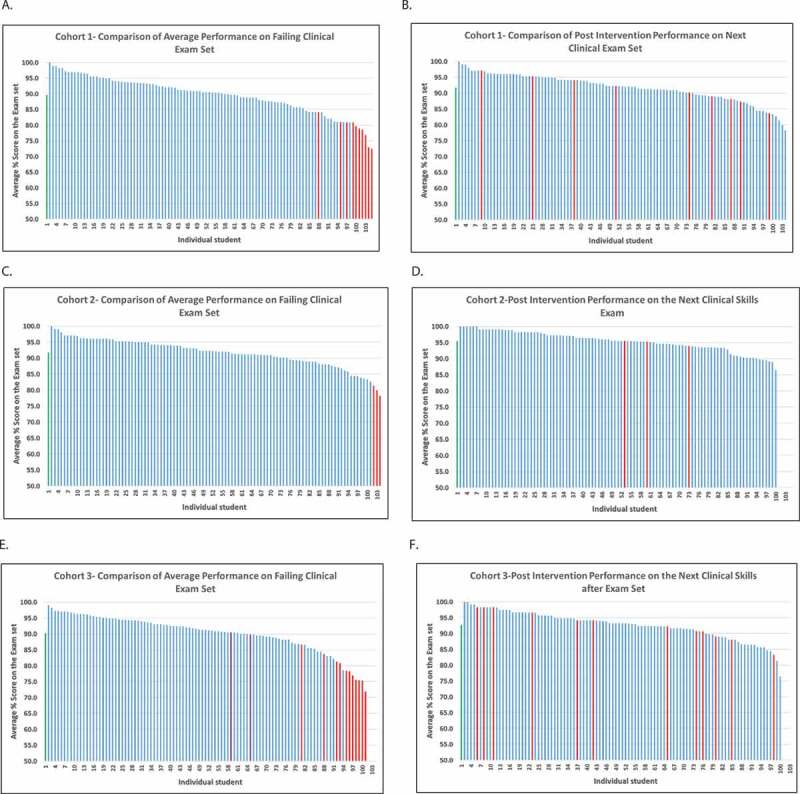
The column on the left (A,C and E) shows average scores of all students in the class. Each bar represents the score of one student. Blue bars: students that passed the clinical skills exam set. Red Bars: students that failed one or more stations of the clinical skills exam set. The column on the right (B, D and F) shows average scores of all students in the class on the next clinical skills exam set. Red bars: student who failed the previous exam set listed in A and then were remediated using the hybrid model. Green bar: the class average on the exam. The cohort is 103 students normally and the missing bars denote students that deferred the exam for some reason and extra bars represent additional students examined with the cohort

Comparison of remediated students’ scores (average of 3 exam stations) pre- and post-coaching to their cohort’s performance showing sustained improvement on a subsequent clinical skills exam (Figure 1).

**A and B**: Graph A represents student cohort 1, with nine students failing one or more of the skills sets on the exam set (red bars). Graph B represents the majority of failing students from cohort 1 scoring above the class average with the next clinical skills exam set tested, suggesting sustained use of SRL and improved outcome after they remediated with the hybrid model.**C and D**: Graph C depicts student cohort 2 with the red bars representing the students who failed this clinical exam set. Graph D depicts these same students with the red bars who received the hybrid remediation, with the majority scoring above the class average in the next clinical exam set. This also suggests improved outcomes with the hybrid model.**Eand F**: Graph E depicts student cohort 3 with red bars representing students who failed the clinical exam set. The purple bars in this graph represent students who had failed a previous exam and also had been remediated with the hybrid model showing improved performance on this current exam set. Graph F represents the students who failed from Graph E (red bars), after undergoing the hybrid remediation, the majority showing an improved score in the next clinical exam set. Again, this suggests improvement and sustained SRL from the remediation.

## Lessons learned/how it can be adapted

We found a more focused approach combining learning models results in better learning outcomes. We suggest surveying remediated learners in future to assess their insight on the process. Faculty can expect to spend one and a half to two hours on average per student overall. A future consideration is to provide strategies early on to manage common errors, encouraging student self-sufficiency. Less lengthy, more targeted remediation sessions may result.

This is an adaptable approach for programs. Faculty can set parameters for early identification of underperforming students and use a similar hybrid coaching model to support and strengthen student SRL for sustained improved outcomes.

## References

[1] Patel R, Tarrant C, Bonas S, et al. The struggling student: A thematic analysis from the self-regulated learning perspective. Med Educ. 2015;49:417–3. 25800302 10.1111/medu.12651

[2] Moon SH, Myung SJ, Yoon HB, et al. Deliberate practice as an effective remediation strategy for underperforming medical students focused on clinical skills: A prospective longitudinal study. J Korean Med Sci. 2019;34. DOI:10.3346/jkms.2019.34.e84 PMC6427051 30914904

[3] Hauer KE, Teherani A, Irby DM, et al. Approaches to medical student remediation after a comprehensive clinical skills examination. Med Educ. 2008;42:104–112. 18042183 10.1111/j.1365-2923.2007.02937.x

[4] Ericsson KA. Acquisition and maintenance of medical expertise: a perspective from the expert-performance approach with deliberate practice. Acad Med. 2015;90(11):1471–1486. 26375267 10.1097/ACM.0000000000000939

[5] Connor DM, Dhaliwal G. When less is more for the struggling clinical reasoner. Diagnosis (Berl). 2015;2:159. 29540031 10.1515/dx-2015-0014

[6] Zimmerman BJ. Becoming a self-regulated learner: an overview. *Theory Into Pract*. 2002;41:64–70.

[7] Sandars J, Cleary TJ. Self-regulation theory: applications to medical education: AMEE Guide No. 58. Med Teach. 2011;33:875–886. 22022899 10.3109/0142159X.2011.595434

[8] Allal L. Assessment and the co-regulation of learning in the classroom. *Assess Educ Princ Pol Pract*. 2019. 10.1080/0969594x.2019.1609411.

[9] Rich VJ. Proposing a model of co-regulated learning for graduate medical education. Acad Med. 2017;92:1100–1104. 28177957 10.1097/ACM.0000000000001583

[10] Bransen D, Govaerts MJB, Sluijsmans DMA, et al. Beyond the self: the role of co‐regulation in medical students’ self‐regulated learning. Med Educ. 2020;54:234–241. 31788840 10.1111/medu.14018 PMC7065189

